# Temporal changes in gene expression and genotype frequency of the ornithine decarboxylase gene in native silverside *Basilichthys microlepidotus*: Impact of wastewater reduction due to implementation of public policies

**DOI:** 10.1111/eva.13000

**Published:** 2020-06-22

**Authors:** David Veliz, Noemi Rojas‐Hernández, Sylvia V. Copaja, Caren Vega‐Retter

**Affiliations:** ^1^ Departamento de Ciencias Ecológicas Facultad de Ciencias Universidad de Chile Santiago Chile; ^2^ Instituto de Ecología y Biodiversidad (IEB) Universidad de Chile Santiago Chile; ^3^ Núcleo Milenio de Ecología y Manejo Sustentable de Islas Oceánicas (ESMOI) Departamento de Biología Marina Universidad Católica del Norte Coquimbo Chile; ^4^ Departamento de Química Facultad de Ciencias Universidad de Chile Santiago Chile

**Keywords:** freshwater, Maipo River Basin, ornithine decarboxylase, public policies, silverside

## Abstract

Human activity has caused a deterioration in the health and population size of riverine species; thus, public policies have been implemented to mitigate the anthropogenic impacts of water use, watercourse transformation, and pollution. We studied the Maipo River Basin, one of the most polluted with untreated wastewater in Chile, for a period of 12 years (2007‐2019). Since the implementation of new public policies, including the operation of a wastewater collector (2012), the Maipo River Basin is currently much less polluted by untreated water than before. To analyze the impact of wastewater reduction in this river basin, we studied the native silverside (*Basilichthys microlepidotus*), which inhabits both polluted and unpolluted areas of the river basin. Previous studies reported the overexpression of the ornithine decarboxylase (*odc*) gene, heterozygote deficit, and high frequency of a homozygote *odc* genotype in silverside populations that inhabit wastewater‐polluted sites, suggesting a phenotypic change and genotypic selection in response to pollution. Here, a population affected and another population unaffected by wastewater were studied before and after implementing the wastewater collector. The physicochemical data of water samples, changes in *odc* expression and microsatellite variability, and *odc* genotype frequencies were analyzed. The results showed physicochemical changes in the affected site before and after the operation of the wastewater collector. The microsatellite loci showed no changes in either population. The *odc* expression in the affected site was higher before the operation of the wastewater collector. Significant changes in the genotype frequencies of the *odc* gene before and after the wastewater collector operation were detected only at the affected site, wherein the homozygous dominant genotype decreased from >59% to <25%. Our results suggest that public policies aimed at mitigating aquatic pollution can indirectly affect both gene expression and genotype frequencies of important functional genes.

## 1 INTRODUCTION

Human activities, such as excessive water use, transformation of watercourses, introduction of exotic species, and pollution (Arthington, Dulvy, Gladstone, & Winfield, 2016; Clavero, Blanco‐Garrido, & Prenda, 2004; Dynesius & Nilsson, 1994; Mora, Metzger, Rollo, & Myers, 2007; Myers, 1995), negatively impact populations inhabiting freshwater systems, affecting their survival and adaptation to new environmental conditions. Both lethal and sublethal effects caused by aquatic pollution (Spehar et al., 1982), such as reduced population size (Dudgeon et al., 2006; Haro et al., 2000), decreased egg fertility and spermatozoid mobility (Kime, 1995), and rapid adaptation to specific pollutants in water (Reid et al., 2016; Whitehead, Clark, Reid, Hahn, & Nacci, 2017), have been documented in the literature.

Although most studies have focused on the evidence of impacts caused by human activity (e.g., Jones & Stanley, 2016), there are also studies reporting the positive effects of changes in environmental policies. One well‐known positive example is the policy to remove reservoirs, allowing the reestablishment of natural watercourses and free movement of minerals and nutrients from the mountains to the sea (Bednarek, 2001). Furthermore, policies related to the reestablishment of a population (Hitt, Eyler, & Wofford, 2012) and recolonization of the fish species upstream of the reservoir have been reported (Catalano & Bozek, 2007). In the case of pollution, returning the systems to their original state is a major challenge for environmental policies. In some rivers, an increase in species richness has been described (Brown et al., 2011; Northington & Hershey, 2006); however, in some cases, the expected reduction in the pollution level was negligible (Schiff & Macbroom, 2011). To our knowledge, no studies have been performed to determine the relationship between pollution reduction and allele or genotype frequency changes in freshwater fish populations, which can be further associated with phenotype and gene expression that ultimately contribute to an organism's fitness (Agashe, Martinez‐Gomez, Drummond, & Marx, 2013; Goudet & Keller, 2002).

During the last decade, we monitored the Maipo River Basin, which is one of the most polluted river basins in Chile. According to the 2017 census, 7 million people (approximately 40% of the Chilean population) live close to this basin. Because of the organic matter present in untreated sewage generated by the growing number of human population and industrial facilities, the Maipo River has experienced water quality deterioration and eutrophication (Pardo, Vila, & Bustamante, 2008; Vega‐Retter, Muñoz‐Rojas, Vila, Copaja, & Veliz, 2014). Furthermore, the Maipo River Basin has the largest number of factories in the country and mining operations in the Andes Mountains, which affect the river's flora and fauna. A previous study on fish diversity in this river basin has revealed a significant reduction in species richness and abundance over the last 30 years (Muñoz, 2007). The Mapocho River, a tributary of the Maipo River that crosses the city of Santiago, has been receiving untreated wastewater since the city was established. At the end of the 19th century, the pollution in the Mapocho River increased because of human migration from the countryside to the city and incipient industrialization (Castillo‐Fernández, 2018). However, in 2009, new environmental policies that directly channeled wastewater to the water treatment plants in Santiago City were implemented. The channel used for this purpose was constructed in 2012 (Superintendent of Sanitary Systems, https://www.siss.gob.cl/586/w3‐article‐7733.html).

The limnetic neotropical silverside (*Basilichthys microlepidotus*, Atheriniformes: Atherinopsidae), an atherinid endemic to Chile, inhabits lakes and rivers from the Copiapó River (27°22′S) to the Chiloé Island (41°52′S) (Veliz et al., 2012). It is a microphagous species that feeds on insect larvae, small invertebrates, filamentous algae, and detritus (Bahamondes, Soto, & Vila, 1979; Duarte, Feito, Jara, Moreno, & Orellana, 1971). *Basilichthys microlepidotus* has a 1‐year generation time, with a reproductive period ranging from August to January (Comte & Vila, 1992). This species is considered vulnerable based on its conservation status (Ministerio de Medio Ambiente Chile, 2018). The *B. microlepidotus* populations inhabiting polluted and unpolluted sites in the Maipo River Basin have been described previously (Vega‐Retter et al., 2014). In particular, an RNA‐seq analysis revealed that the expression of three cancer‐related genes in fish inhabiting polluted areas was higher than those inhabiting unpolluted areas (Vega‐Retter et al., 2018). One of these genes, ornithine decarboxylase (*odc*), which is related to tumor development and progression, showed synonymous mutations and evidence of change in genotype frequencies, such as heterozygote deficit and increased frequency of a homozygote genotype, in polluted areas. Further, Vega‐Retter et al. (2014) reported that pollution did not affect its neutral genetic diversity (microsatellite loci), while Vega‐Retter, Vila, and Véliz (2015) hypothesized that pollution is potentially implicated in genotype selection. It is important to note that these results were based on the samples analyzed before the construction of the wastewater collector (Vega‐Retter et al., 2018). Therefore, this system is a suitable model to determine the effects of pollution reduction on genotype selection.

Thus, owing to the significance of the *odc* gene in species health and substantial changes in genotype frequencies, we hypothesized that *odc* expression and genotype frequency in silverside may be affected by the operation of the wastewater collector. In this study, we investigated a polluted site, Melipilla (MEL), located downstream of the Maipo and Mapocho River junctions, and an unpolluted site (control site), San Francisco de Mostazal (SFM), located upstream of the Maipo River and uninfluenced by the Mapocho River. From these sites, we obtained the following data: (a) basic physicochemical properties of water at both polluted and unpolluted sites, (b) change in *odc* expression levels of fish liver sampled in 2012 (before) and 2016 (after), and (c) *odc* genotype frequencies before (2007 and 2011) and after (2018 and 2019) the wastewater collector operation. In addition, the variability of eight microsatellite loci used as the control loci in the same sites was used to describe possible changes in neutral loci variability over time.

## 2 MATERIALS AND METHODS

### 2.1 Sampling sites and sample collection

**FIGURE 1:**
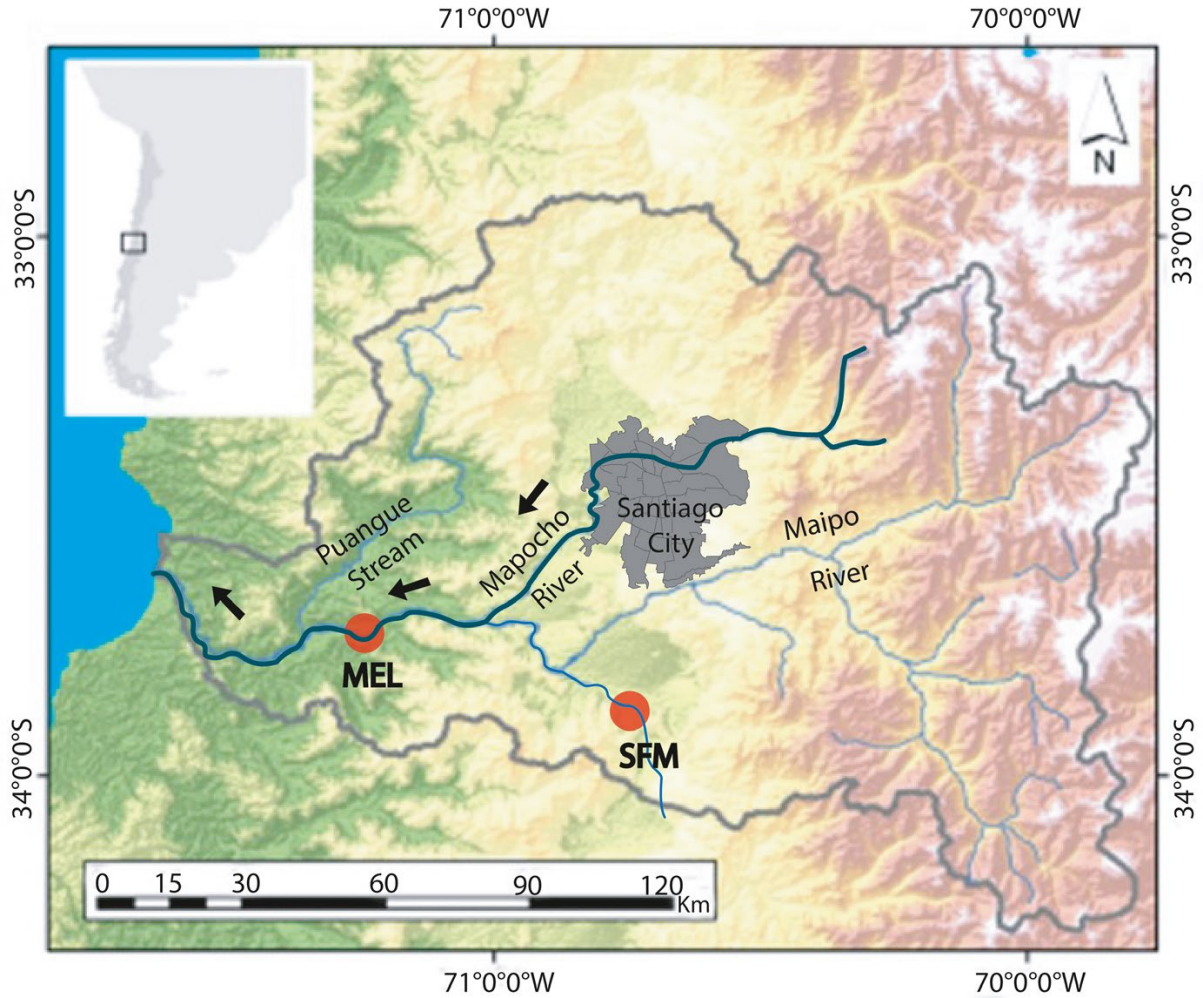
Sampling sites in the Maipo River Basin. MEL—Melipilla (affected site), SFM—San Francisco de Mostazal (unaffected site)

**FIGURE 2:**
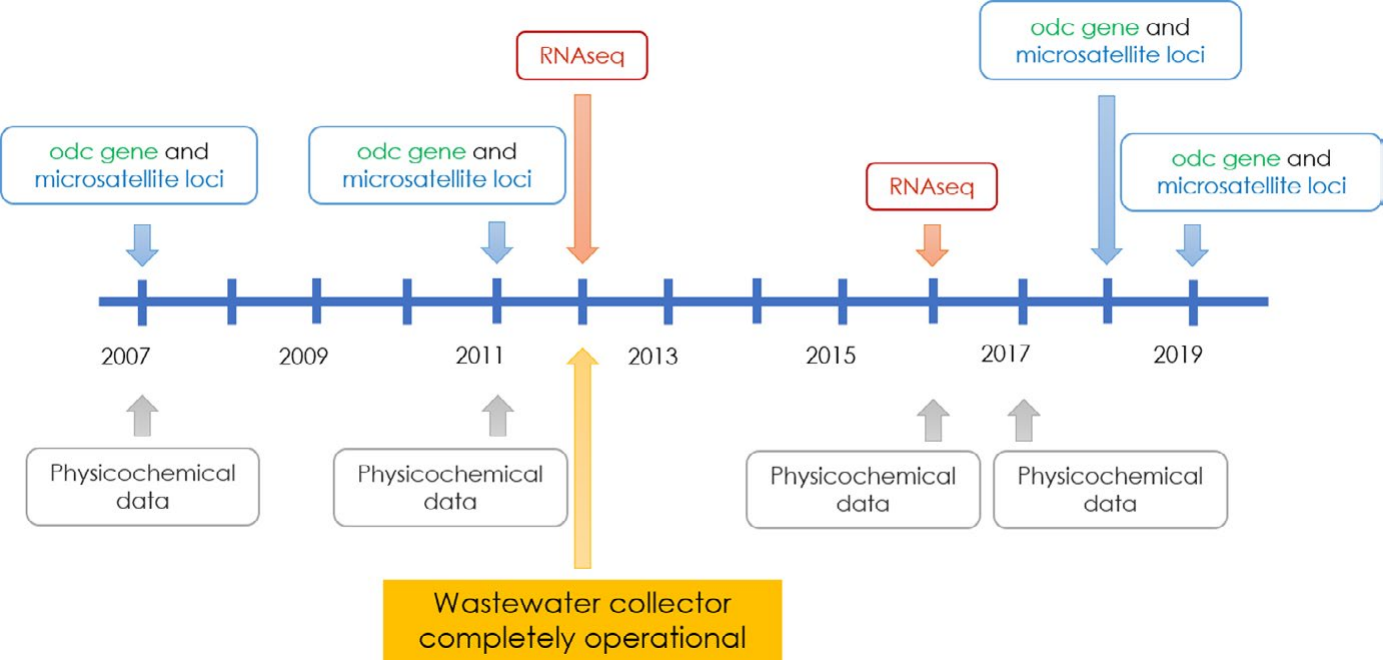
Sampling timeline. The figure shows date of water collection for physicochemical analysis and fish sampling for RNA‐seq, microsatellite, and *odc* analyses before and after the wastewater collector operation

Samples were obtained from two sites in the Maipo River Basin: one affected (MEL) and one unaffected (SFM) by the Mapocho River wastewater (Figure 1). To determine the changes in the *odc* genotype frequencies, fish samples were collected before (2007 and 2011) and after (2018 and 2019) policy changes in the Maipo River Basin (Figure 2). The samples obtained in 2007 and 2011 were used previously by Quezada‐Romegialli, Fuentes, and Veliz (2010) and Vega‐Retter et al. (2014), respectively. It is important to note that the changes in *odc* expression were estimated using samples collected in 2012 and 2016.

### 2.2 Physicochemical characterization of water from sampling sites

To assess the pollution level at each sampling site, we determined the physical and chemical characteristics of the water samples collected before (2007 and 2011) and after (2016 and 2017) policy changes in the Maipo River Basin. Water physicochemical parameters, including electrical conductivity (EC), pH, and total dissolved solids (TDS), were measured in situ three to six times at each site on each sampling date using a multiparameter device (Hanna Instruments). The nitrate ( ${\text{NO}}_{3}^{-}$ ), ammonium (NH_4_^+^), sodium (Na^+^), potassium (K^+^), calcium (Ca^2+^), and magnesium (Mg^2+^) concentrations were measured in one to six water samples (1 L) from each site and sampling year. Each sample was filtered using a cellulose nitrate filter with a 0.45 µm pore diameter (Sartorius, Göttingen, Germany) according to Clesceri, Greenberg, and Eaton (2005), and ion concentrations were measured by atomic absorption spectroscopy (Shimadzu AA‐6880). To determine the dissolved oxygen (DO) concentration, one to six water samples collected from each site in each sampling year were taken in 200‐mL polycarbonate bottles (Nalgene), fixed with manganese sulfate and alkaline iodide, and analyzed using the Winkler method, according to Strickland and Parsons (1968). To analyze the data, three principal component analyses (PCAs) were performed with the function prcomp and fviz_pca_biplot of the library factoextra (Kassambara & Mundt, 2017) in the R software (R Core Team, 2019). To qualitatively detect the relationships among the collection sites and sampling years, the first PCA was performed with all data, including both sampling sites and all sampling years. The second and third PCAs were performed with MEL and SFM separately in order to observe the physicochemical changes that have occurred at each site in each sampling year.

### 2.3 Change in *odc* expression over time

To quantify the expression of the *odc* gene, the RNA‐sequencing (RNA‐seq) data of the samples collected in 2012 and 2016 were used. In both years, the analysis was performed on individuals collected from four sites. In the present study, we used data from two of those sites: MEL and SFM sites. The liver RNA samples of the three silversides collected from each sampling site in 2012 were subjected to RNA‐seq using the Ion Torrent™ sequencing platform (Life Technologies) (Vega‐Retter et al., 2018; Vega‐Retter & Véliz, 2014). The raw data from the samples were assembled de novo using the Mira Assembler (Cheveruex, Wetter, & Suhai, 2009). On the other hand, for the samples collected in 2016, RNA‐seq was performed on 12 individuals, six per sampling site. Total RNA of these 12 samples was extracted using the PureLink™ RNA Mini Kit (Ambion, Life Technologies). RNA quality and quantity were determined using the Agilent 2100 Bioanalyzer (Agilent Technologies) at Genoma Mayor Sequencing Services (Santiago, Chile). Samples with a RIN > 7 were sequenced using the Illumina HiSeq 4000 sequencer (Illumina), and the subsequent bioinformatics analysis was also performed at Genoma Mayor Sequencing Services.

Raw reads with a mean Phred score of <Q30, ambiguous bases >10%, and reads < 50 bp were removed using the software programs Trim Galore (https://www.bioinformatics.babraham.ac.uk/ projects/trim_galore/), Cutadapt (Martin, 2011), and prinseq‐lite.pl script (http://prinseq.sourceforge.net/manual.html). The de novo assembly of the reads was performed using the Bridger software (Chang et al., 2015) with the default parameter settings: *k*‐mer length = 25, seed *k*‐mer minimum coverage = 2, and seed *k*‐mer minimum entropy = 1.5. In this assembly, the detection of isoforms was not considered, and contigs > 200 bp were retained for further analyses. The CD‐HIT software (http://weizhongli‐lab.org/cd‐hit/) was used to create a set of nonredundant contigs with a similarity threshold of 80%. The contigs were annotated with Blastx against the UniProtKB/Swissport database (http://www.uniprot.org/) with a cutoff e‐value of 1E‐5 and mapped against the reference sequence using the Bowtie2 software (Langmead & Salzberg, 2012). RNA‐sequencing of the samples collected in 2012 and 2016 resulted in different numbers of reads; thus, the number of reads mapped to the *odc* contig of each individual was normalized considering the total number of reads. For both sampling times, the ratio of the change in *odc* expression between the affected (MEL) and unaffected (SFM) sites was used to determine possible changes over time.

### 2.4 Sample collection for *odc* gene sequencing and microsatellite genotyping

To detect changes in *odc* genotype frequencies over time, 21–24 silverside individuals were collected each from the affected (MEL) and unaffected (SFM) sites using a low‐impact electrofishing device (Samus, Poland) in 2007, 2011, 2018, and 2019. Owing to their small size (<14 cm), the collected silversides were sedated with tricaine methanesulfonate (MS‐222) at a concentration (12 mg/L) lower than that proposed by Topic Popovic et al. (2012). A small piece of the caudal fin was dissected and stored in 95% ethanol. To reduce the effect of anesthesia, the fish were maintained in clean and oxygenated water for 20 min before being released back into the river. The sample collections were conducted in compliance with Chile's existing laws (Resolución Exenta No. 1725, 2887, 3078, and 3329; Subsecretaria de Pesca). Genomic DNA was isolated using the salt extraction method (Aljanabi & Martinez, 1997) and then used for the amplification of the *odc* gene and microsatellite loci.

### 2.5 Amplification of microsatellite loci

Eight microsatellite loci were used as a control neutral loci, including four (Odon02, Odon07, Odon09, and Odon39) described by Beheregaray and Sunnucks (2000) for *Odontesthes perugiae* and four (Odon01, Odon19, Odon59, and Odon71) described by Koshimizu et al. (2009) for *O. bonariensis*. The PCR conditions used were replicated from Muñoz, Quezada‐Romegialli, Vila, and Veliz (2011). For Odon02, Odon07, Odon09, and Odon39, the amplification was carried out in a 10‐µl reaction volume containing 1.3 µl of 10X PCR buffer (Invitrogen), 0.5 µl of MgCl_2_ (50 mM) (Invitrogen), 0.5 µl 50 ng/µL forward and reverse primers (Applied Biosystems, Foster City, CA, USA), 2.4 µl 2.5 mM dNTPs (Invitrogen), 0.5 µl 10 mg/ml BSA, 4.38 µl H_2_O, 0.1 U of *Taq* polymerase (Invitrogen), and 2 µl 50 ng/ml genomic DNA. The cycling conditions for touch‐down PCR consisted of an initial denaturing step at 94°C for 3 min, followed by 5 cycles at 94°C for 20 s, specific starting annealing temperature (decreasing 2°C per cycle) for 45 s, 72°C for 60 s, followed by 27 cycles at 94°C for 20 s, specific final annealing temperature for 60 s, 72°C for 60 s, and a final elongation step at 72°C for 4 min. The decreasing temperatures for Odon02 and Odon07 ranged from 61 to 53°C and 55 to 47°C, respectively, while those for Odon09 and Odon39 ranged from 59 to 51°C. For Odon01, Odon19, Odon59, and Odon71, the amplification was carried out in an 18‐μL reaction volume containing 10 µl of Type‐it Microsatellite PCR Master Mix (QIAGEN. Hilden, Germany), 5 µl of H_2_O, 0.5 µl 50 ng/µl forward and reverse primers, and 2 µl 50 ng/µl genomic DNA. The cycling conditions consisted of an initial denaturing step at 95°C for 3 min, followed by 35 cycles at 95°C for 30 s, 60°C for 30 s, 72°C for 1 min 30 s, and a final elongation step at 72°C for 5 min. The PCR products were sequenced by the sequencing service of the Pontificia Universidad Católica de Chile. The allelic matrix was generated using the GeneMarker software (SoftGenetics). The number of alleles per locus and the expected (He) and observed (Ho) heterozygosities were estimated using the Genetix software (Belkhir, Borsa, Chikhi, Raufaste, & Bonhomme, 2000). In the same software, the *F*_IS_ index with 5,000 iterations was used to test for departures from Hardy–Weinberg equilibrium (HWE), while the *F*_ST_ index with 1,000 permutations was used to analyze the temporal changes in allelic frequencies.

### 2.6 Reciprocal gene flow between sampling sites

To estimate the contemporary reciprocal migration between the MEL and SFM sites in each sampling year, we used the microsatellite loci and the program BAYESASS 3.0 (Wilson & Rannala, 2003) with a burn‐in period of 3,000,000 iterations, after burn‐in of 30,000,000 iterations with sampling at each 100 iterations. The mixing parameters for allele frequencies, migration rates, and inbreeding coefficients were defined as 0.5, 0.3, and 0.7, respectively. Five independent runs were conducted, starting with different random seeds, and the results were expressed in terms of the average value of these five independent runs.

### 2.7 Changes in the *odc* genotype frequency over time

To detect changes in genotype frequencies over time, the *odc* genes from the fish sampled in 2007, 2011, 2018, and 2019 from the MEL and SFM sites were sequenced. The primers and PCR conditions used were replicated from Vega‐Retter et al. (2018). The 25‐μl PCR mixture contained 1X PCR buffer (Invitrogen), 3.2 nM MgCl_2_ (Invitrogen), 5 pmol 50 ng/L forward and reverse primers (Applied Biosystems), 0.2 U/μl 2.5 mM dNTPs (Invitrogen), 0.1 U Platinum *Taq* polymerase (Invitrogen), and 1.5 μl 65 ng/μl template DNA. The PCR cycling profile consisted of a denaturing step at 94°C for 3 min, followed by 30 cycles at 94°C for 30 s, 60°C for 90 s, and 72°C for 90 s, and a final elongation step at 72°C for 10 min. The PCR products were examined by electrophoresis on 1.5% agarose gels and sent for forward and reverse sequencing. The samples from 2011 were sequenced by Macrogen Inc. (Korea), while the samples from 2007, 2018, and 2019 were analyzed by the sequencing service of the Pontificia Universidad Católica de Chile. Polymorphic sites from the two sequencing results were identical.

Vega‐Retter et al. (2018) reported the presence of three synonymous mutations in the sequences; thus, the phase method in the DnaSP v.4.9 software (Rozas et al., 2017) was used to detect the correct sequence of each allele. Appendix S1 shows the Sanger DNA sequence of each mutation detected in this study. The number of alleles per locus and the expected (He) and observed (Ho) heterozygosities were estimated using Genetix software (Belkhir et al., 2000). The *F*_IS_ index with 5,000 allele permutations was used to test for departures from the HWE, and the temporal changes in allele frequencies were estimated using the F_ST_ index with 1,000 permutations in the Genetix software. To detect temporal changes in genotype frequencies, which are a response variable to the presence of the wastewater collector, odc genotypes were classified into four categories: (a) homozygote GTCGC/GTCGC or the genotype with higher frequency before the operation of the collector at the affected site, (b) heterozygote with the GTCGC and other alleles, (c) all other homozygotes lacking the GTCGC allele, and (e) all heterozygotes lacking the GTCGC allele. These were analyzed using Fisher's exact test implemented in the R software (R Core Team, 2019). Using the results of the above‐mentioned analyses, we tested for statistically significant changes in allelic or genotype frequencies over time in silversides collected from both affected and unaffected sites.

Genotype frequencies were used to estimate the fitness values for each genotype at each site (MEL and SFM). The fitness values were calculated based on the ratio between the genotype frequency values at two different time periods (as in Veliz, Bourget, & Bernatchez, 2004). From each sampling site, we considered the frequencies of each genotype before (2007, 2011) and after (2018, 2019) the operation of the wastewater collector. Thus, we obtained four fitness values per genotype and site (2007 versus 2018; 2007 versus 2019; 2011 versus 2018, and 2011 versus 2019). Therefore, the fitness component considered here corresponds to genotype survival from before to after the operation of the wastewater collector. To avoid zero values in the denominator, we pooled the data of the homozygote and heterozygote without the GTCGC allele. The fitness values of the genotypes from the same sampling site were compared using the independent 2‐group Mann–Whitney *U* test implemented in the R software.

## 3 RESULTS

### 3.1 Temporal changes in the physicochemical features of water

**FIGURE 3:**
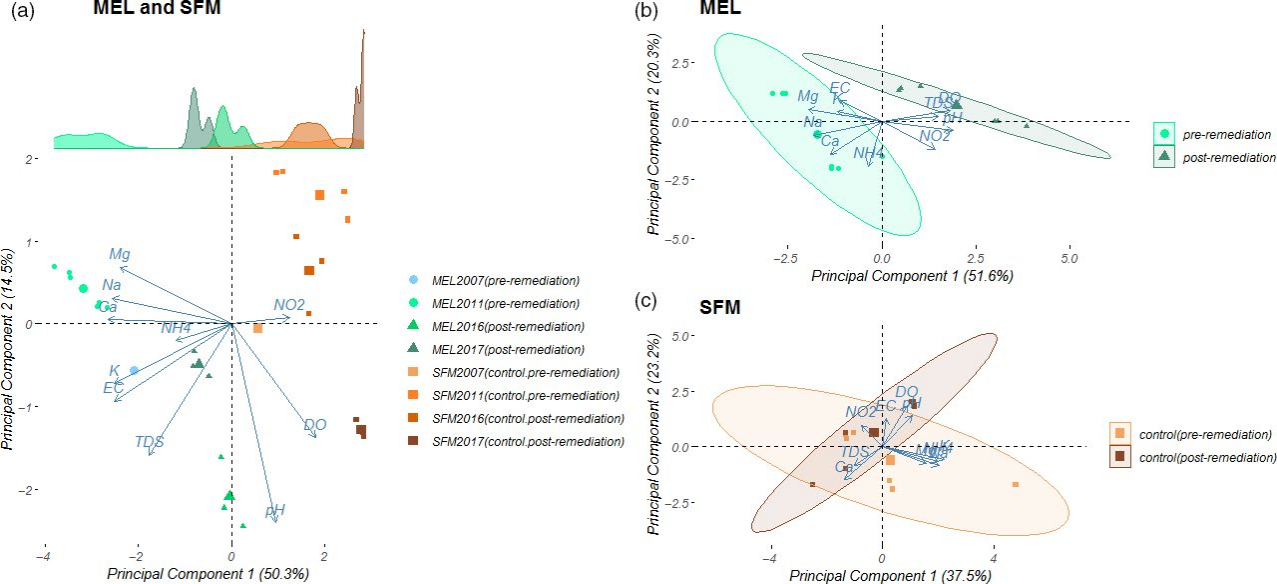
Principal component analysis of the 9 environmental variables measured for four years (2007, 2011, 2016, and 2017) in the affected (MEL) and the unaffected (SFM) sites. (a) PCA including data of both MEL and SFM. Density frequencies for the score data by site and date area above the biplot. (b) PCA with pooled data from MEL pre‐ and postremediation. (c) PCA with pooled data from SFM pre‐ and postremediation. Largest symbols in the biplot represent the centroids

The first PCA performed with the data from both MEL and SFM sites accounted for 64.8% of the total variance (Figure 3a). PC1 (50.3% of the total variance; eigenvalue = 5.03) was mainly correlated with five variables showing high loadings (*L*), specifically Ca (*L* = 0.40), Na (*L* = 0.39), EC (*L* = 0.38), DO (*L* = −0.27), and TDS (*L* = 0.27), while PC2 (14.5% of the total variance; eigenvalue = 1.45) was mainly correlated with pH (*L* = −0.68). PC1 clearly segregated the data obtained from MEL and SFM. For example, the MEL samples had higher mean EC values than the SFM samples. A similar pattern was also observed for the DO values (see Appendix S2). However, after remediation, the physicochemical values of MEL were found to approach the values of the control site (SFM).

Furthermore, the data of both MEL and SFM were analyzed separately using two different PCAs. For MEL, it was possible to observe a spatial segregation of data before and after remediation (Figure 3b, PC1 = 51.9% of the variance, eigenvalue = 5.19; PC2 = 19.9% of the variance, eigenvalue = 1.99). PC1 was mainly correlated with EC (*L* = 0.25), pH (*L* = −0.40), TDS (*L* = −0.30), DO (*L* = −0.39), Na^+^ (*L* = 0.39), Ca^2+^ (*L* = 0.29), Mg^2+^ (*L* = 0.39), while PC2 was correlated with ${\text{NH}}_{4}^{+}$ (*L* = 0.65); however, the PCA in the control site (SFM) showed an overlap of data before and after remediation in both components (Figure 3c, PC1 = 37.6% of variance, eigenvalue = 3.75; PC2 = 23.2% of variance, eigenvalue = 2.32) (Figure 3c). These results show changes in physicochemical features before and after remediation only at the affected site (MEL). A summary of the physicochemical data is shown in Appendix S2.

### 3.2 Changes in *odc* expression over time

**FIGURE 4:**
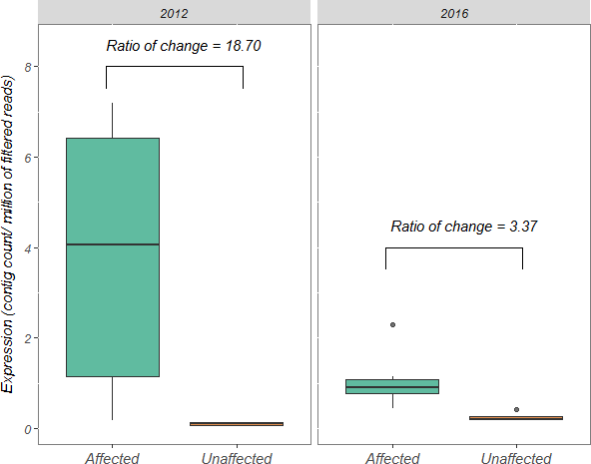
Boxplot of the standardized gene expression per million of filtered reads of the *odc* gene in the individuals sampled in 2012 and 2016 from the affected (MEL) and the unaffected (SFM) sites. Points represent outlier data (expression 1.5% greater than the interquartile range [IQR])

The RNA‐seq data from 2012 and 2016 were used to compare the expression of the *odc* gene before and after implementation of the wastewater collector. A total of 30.15 million reads were obtained from the silversides sampled in 2012. After trimming, a total of 17.28 million reads were retained for mapping data of MEL and SFM. The reads per sample ranged from 1.83 to 4.7 million. The de novo assembly generated 34,321 nonredundant contigs longer than 200 bp, with an N50 value of 674. For the silversides sequenced in 2016, a total of 190.66 million reads were obtained after trimming data of MEL and SFM. The reads per sample ranged from 11.89 to 18.74 million. The de novo assembly generated 63,425 nonredundant contigs longer than 200 bp, with an N50 of 2,889. The contigs annotated for the *odc* gene had lengths of 1958 and 1,368 bp in the fish sampled in 2012 and 2016, respectively. As shown in Figure 4, the normalized expression values in SFM were lower than those in MEL in both sampling years. Moreover, the *odc* expression levels were higher in MEL silversides sampled in 2012 than in those sampled in 2016. Furthermore, the ratio of change estimated between SFM and MEL silversides sampled in the same year was significantly higher before (18.7) than after (3.37) the collector was operational.

### 3.3 Microsatellites as control loci and gene flow

The summary statistics for the microsatellite loci studied are shown in Appendix S3. Two monomorphic loci (Odon59 and Odon39) were excluded from further analysis. The results showed no significant departures from HWE at any sampling site or in any sampling year. Furthermore, the *F*_ST_ index showed no significant difference between the sampling years at each site (Table 1). The current reciprocal migration estimated between the affected (MEL) and unaffected (SFM) sites for different sampling years showed self‐recruitment values >74% with migrants between sites ranging from 13% to 26% (Table 2).

**TABLE 1:** Matrix of pairwise *F*_ST_ estimate with microsatellites for MEL (above the diagonal) and SFM (below the diagonal) between sampling dates

	2007	2011	2018	2019
2007		0.0076	0.0038	0.0069
2011	0.0115		0.0083	0.0197
2018	−0.0077	0.0014		−0.0113
2019	0.0125	0.0176	0.0017	

Note

No significant differences were found.

**TABLE 2:** Migration rate estimated for *B. microlepidotus* in MEL and SFM sites in each sampling year

	2007	2011	2018	2019
SFM to MEL	0.248 (0.002)	0.133 (0.003)	0.129 (0.003)	0.129 (0.003)
MEL to SFM	0.213 (0.001)	0.287 (0.003)	0.266 (0.003)	0.266 (0.003)

Note

Each value represents a mean of five different runs. Standard deviations are shown in parentheses.

### 3.4 Changes in *odc* variability over time

A total of six *odc* alleles were identified in all samples. The *F*_IS_ analyses revealed no significant departures from HWE for all samples, except for the MEL samples (affected site) obtained in 2011 (*F*_IS_ = 0.515, *p* = .005). Notably, the MEL samples collected in 2007 showed the second highest *F*_IS_ value (*F*_IS_ = 0.26108, *p* = .131) compared to the samples collected in other years (Appendix S4). No significant differences (*p* > .05) in the *F*_ST_ values of the control (SFM) samples were observed between different sampling years (Table 3). In contrast, significant differences were observed in all MEL samples (*p* < .05), except for the comparison between the years before (2007 versus 2011) and after (2018 versus 2019) the operation of the wastewater collector.

**TABLE 3:** Matrix of pairwise *F*_ST_ estimate with the *odc* gene variability for MEL (above the diagonal) and SFM (below the diagonal) between sampling dates

	2007	2011	2018	2019
2007		−0.0128	0.0661*	0.1319*
2011	−0.0034		0.0625*	0.0904*
2018	0.0001	0.0057		0.0383
2019	−0.0106	−0.0052	0.0321	

* *p*‐value < .05.

**FIGURE 5:**
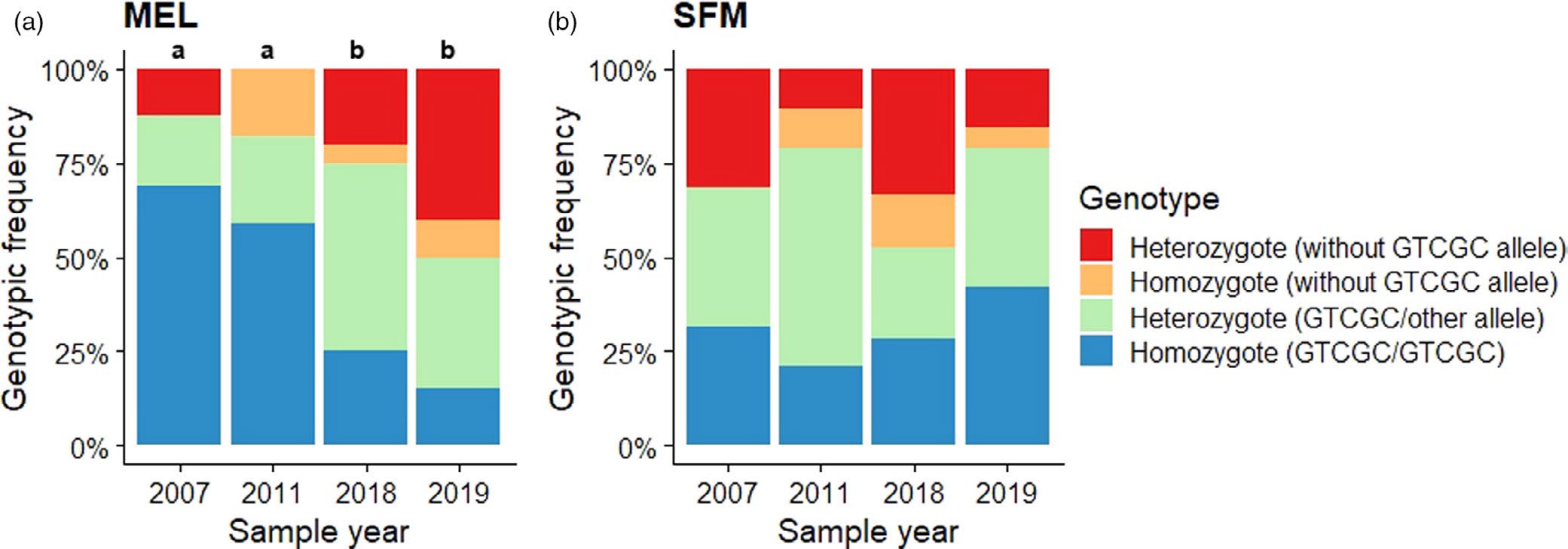
Genotype frequencies observed in silversides collected during different sampling dates from the (a) affected (MEL) and (b) the unaffected (SFM) sites. Bars differing in letters represent statistical difference (*p* < .05), and for panel (b), nonsignificant differences were detected

An analysis of genotype frequencies clearly demonstrated that changes occurred in the MEL samples before and after the operation of the wastewater collector. A Fisher's exact test revealed significant differences in all MEL samples, except for the comparison between the years before (2007 versus 2011) and after (2018 versus 2019) the operation of the wastewater collector. In contrast, no significant temporal changes in the genotype frequencies were observed for the control site (SFM) samples (Table 4, Figure 5).

**TABLE 4:** Fisher exact test *p*‐values for the comparison of the *odc* genotype frequencies in time within the affected (MEL, above the diagonal) and the unaffected (SFM, below the diagonal) site

	2007	2011	2018	2019
2007		0.2026	0.0404*	0.0071*
2011	0.1670		0.0229*	0.0029*
2018	0.4100	0.1455		0.5135
2019	0.5915	0.4693	0.3737	

* *p*‐value < .05.

**FIGURE 6:**
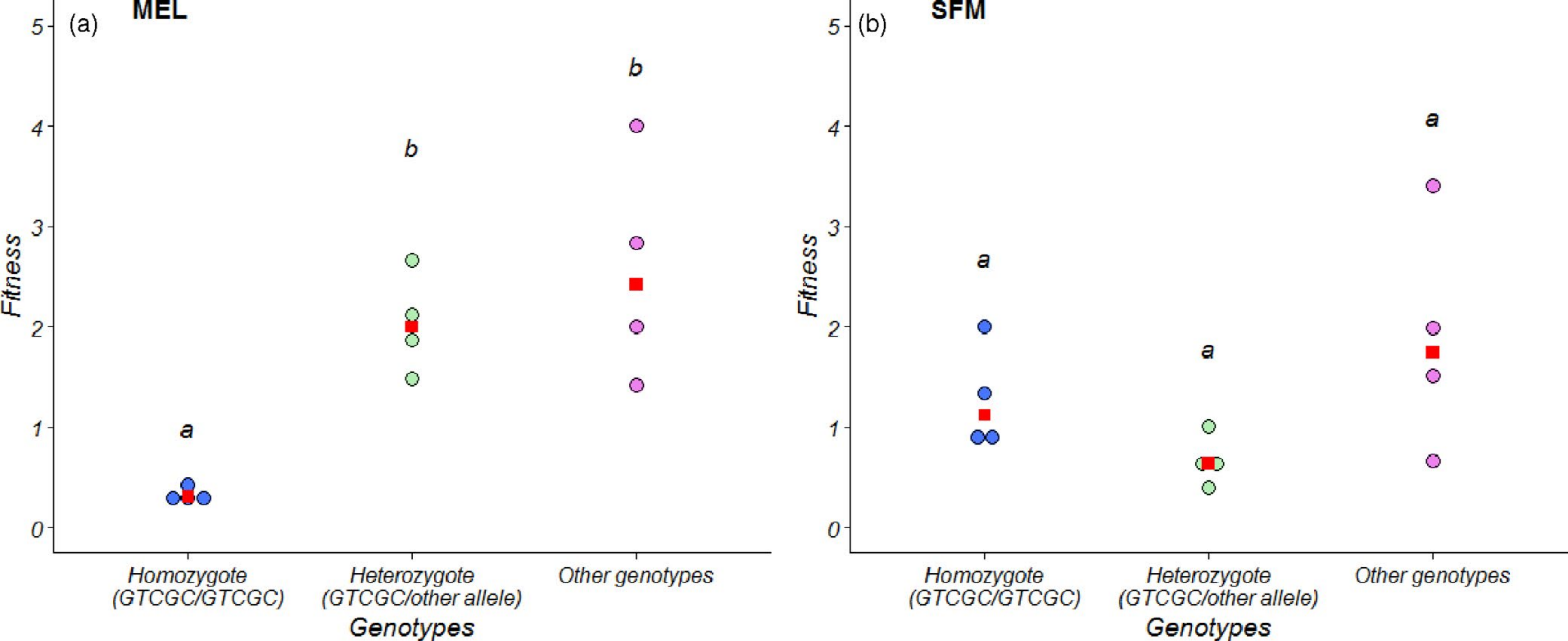
Fitness values for the odc genotypes in (a) MEL (affected site) and (b) SFM (unaffected site). The genotype Other represents all homozygote and heterozygote without GTCGC allele. Genotype sharing the same letter is not significantly different (*p* > .05). Red points represent the median values

The temporal changes in the genotype frequencies of the MEL and SFM silversides are shown in Figure 5, which distinctly shows three important features: (a) the GTCGC/GTCGC homozygote had a high frequency in MEL silversides before (>59%) the collector was operational, (b) the genotype frequencies of the MEL silversides sampled in 2018 and 2019 were similar to the control SFM silversides, and (c) the Fisher's exact test revealed no significant changes in the genotype frequencies of the SFM silversides sampled over the years (*p* < .05). The genotype frequency changes were also used to estimate differences in the fitness of genotypes. These estimations revealed a contrasting pattern between the affected and unaffected sites (Figure 6). The fitness values in the affected MEL site differed mainly from the GTCGC/GTCGC homozygote. The fitness of the GTCGC/GTCGC homozygote (median = 0.31) was statistically lower than the fitness of the heterozygous genotype GTCGC/other allele (median = 1.99) (*W* = 0; *p* = .028) and other alleles (median = 2.41) (*W* = 0, *p* = .028). The genotype GTCGC/other allele showed no statistical differences with the genotype other alleles (*W* = 6; *p* = .686) (Figure 6). In contrast, no significant differences in fitness were observed between the *odc* genotypes in the unaffected SFM sampling sites.

## 4 DISCUSSION

The main objective of this study was to correlate the *odc* expression and genotype frequencies of silversides to the reduction in pollution due to changes in public policies implemented to mitigate water pollution in the Maipo River Basin. The results showed apparent changes in the *odc* expression and genotype frequencies of silversides after the operation of the wastewater collector.

Field studies performed in natural environments have shown that domestic and industrial pollutants affect the natural populations. Domestic wastewater primarily contains microorganisms, degradable organic materials (feces, detergents, oil, and solvents), nutrients, and metals (Hence & Comeau, 2008), while industrial wastewater mainly contains pulp, paper, textiles, chemicals, pharmaceuticals, petroleum, tannery substances, and toxic synthetic and natural substances (Nath, Chakraborty, & Bhattacharjee, 2014).

In the case of the Maipo River Basin, we have no data on the changes in specific pollutants before and after the operation of the wastewater collector. However, EC and DO were among the important variables that explained the differences observed before and after the wastewater collector operation. EC is useful as a general measure of source water quality (Spellman, 2014). A natural system tends to have a relatively constant range of EC; thus, changes in this variable correlate with changes in water quality. Significant changes in conductivity may indicate the likely presence of a discharge or another source of pollution (Spellman, 2014). DO has a negatively proportional relationship with pollution, such that as the DO values decrease, the pollution levels increase (e.g., Sánchez et al., 2007). Overall, we acknowledge that our chemical analyses were limited to only nine elements and do not necessarily represent all types of pollutants; thus, the physicochemical data were used as supporting evidence to understand the changes at the affected site over time.

Previous studies have attempted to associate wastewater with health in both humans and wildlife, suggesting an increased risk of cancer in wastewater treatment workers when compared to national risk averages (Hansen, Hilden, Klausen, & Rosdahl, 2003; Lafleur & Vena, 1991; Lagorio, de Santis, & Comba, 1996; Massaquoi et al., 2015). The effects of wastewater on the physiology and health of freshwater fishes have also been described. Liney et al. (2006) exposed the juveniles of *Rutilus* to wastewater, which resulted in male feminization, alteration of the gonads, and alterations in the tubular diameter of the hepatic ducts, among others. In general, water contamination has also been related to the presence of some neoplasms (Black, 1983; Lauren et al., 2010; Sonstegard, 1977) and papillomas in fish (Grizzle, Melius, & Strength, 1984).

Despite the significance of pollution as one of the primary factors affecting the freshwater diversity (Vörösmarty et al., 2010), studies correlating the selection of specific alleles or genotypes to pollution are limited. For example, the selection of *CYP1A* alleles has been described for *Fundulus heteroclitus* (Williams & Oleksiak, 2011) and *Poecilia reticulata* (Hamilton, Rolshausen, Uren Webster, & Tyler, 2017) inhabiting contaminated areas. Williams and Oleksiak (2011) detected *CYP1A* single nucleotide polymorphisms under selection in three independent *F. heteroclitus* populations inhabiting contaminated sites, suggesting the presence of mechanisms of resistance to different pollutants in the Atlantic coastal waters of the United States.

In the case of the Chilean silverside, Vega‐Retter et al. (2018) studied the genotype frequency changes between polluted and nonpolluted sites detecting: (a) high frequency of the GTCGC/CTCGC homozygote genotype and significant departures from HWE associated to a deficit of heterozygotes and (b) decreased number of heterozygotes, most of which contain the GTCGC allele. In the present study, we complemented this study by detecting high GTCGC/CTCGC homozygote frequency in silversides inhabiting the area affected by wastewater before the operation of the wastewater collector, but it decreased to approximately 25% after the wastewater collector operation. Further, the contrasting fitness values observed in both sites indicated that directional selection acted mainly at the affected site (MEL). In this analysis, the differential fitness found between genotypes at the affected site suggested that it was caused by the spillage of wastewater in the river. Considering these results, our data suggest that the GTCGC allele could confer some kind of resistance to pollution in individuals with this allele. Our results also suggest a relaxation of this selection after the operation of the wastewater collector, with a return to the genotype frequencies observed in the control site, probably due to continuous gene flow between SFM and MEL. Overall, these results indicate that both the *odc* gene and its regulatory region are under selection, but laboratory experiments are needed to support this hypothesis.

Moreover, the present study provided supporting evidence that *odc* expression can be correlated with pollution because the only significant change in the Maipo River Basin between 2012 and 2016 was the operation of the wastewater collector. Vega‐Retter et al. (2018) also studied *odc* gene expression, but only before the wastewater collector operation, and they used two polluted sites that showed high *odc* gene expression. Unfortunately, the second polluted site was not sampled in 2007. To our knowledge, no studies have correlated gene expression with a decrease in wastewater pollution as a result of the implementation of public policies to mitigate aquatic pollution.

In particular, ornithine decarboxylase is the first rate‐limiting enzyme in polyamine biosynthesis that catalyzes the decarboxylation of l‐ornithine to putrescine. The polyamine biosynthetic pathway is a critical regulator of cell growth (Jhingran et al., 2008) and plays an important role in neoplastic transformation and tumor growth (Pegg, 1988). Furthermore, its enzymatic activity has been used as a biological marker for evaluating tumor growth and aggressiveness (Deng et al., 2008). Sato et al. (2018) demonstrated that *odc* plays an important role in the reduction of methylmercury toxicity in mice, while Zahn et al. (1981) reported high *odc* activity and polyamine production in sponges exposed to benzo[α]pyrene. However, field and laboratory studies have shown contrasting *odc* expressions in the presence of pollutants. High level of *odc* activity was observed in fetal and newborn rat brain exposed to hypoxic periods and the pollutant carbon monoxide (Packianathan, Cain, Stagg, & Longo, 1993). Other studies have shown low *odc* activity in *Fondulus heteroclitus* inhabiting sites contaminated with different pollutants, mainly polychlorinated biphenyls (PCBs) and halogenated aromatic hydrocarbons (Oleksiak, 2008). Taking into account the biological importance of *odc* and considerable reduction in its expression at the same location after wastewater reduction, it is important to follow its expression progression over time using real‐time PCR (e.g., Rojas‐Hernandez, Véliz, & Vega‐Retter, 2019). On the other hand, it is important to note that our study was a field work in which the factors affecting *odc* expression could not be treated separately. Thus, further studies in controlled conditions, where the factor of interest is isolated from other factors, such as temperature water regime, parasites, etc., are required. In addition, it is necessary to perform expression analyses of each genotype under different pollution conditions to establish the complete relationship between selection and gene expression.

The results of our study suggest that the implementation of public policies enhanced the water quality of the Maipo River Basin, resulting in a significant increase in the habitat quality of native silversides. However, our findings do not imply that the Maipo River Basin has returned to its original state because only one gene that responds to a specific type of pollutant was studied; thus, a specific study of fish health and population size is needed to confirm that the changes detected here also evidence a recovery of the populations.

BOX 1 **Personal reflection**

I joined Bernatchez Lab in 2000 after 9,000 km travel from Chile. I remember two phrases of Louis during my first years in Québec: “the limit is only in your mind” and “take care.” The first was when I known that Paul Schmidt and David Rand were working with the same species, same loci, and similar hypothesis. I was frustrated by this fact; however, Louis supported me in focusing my PhD thesis on something that was finally great. During this time was also important the support of Edwin Bourget (co‐advisor) and Pierre Duchesne (third unofficial advisor). I learned with them that it is necessary to work hard to achieve our goals, an important lesson for both personal and academic growth. The second phrase was during the summer sampling when the lobster war exploded in one of the sampling sites in New Brunswick. This was another big history never completely told. Many thanks Louis and feliz cumpleaños!

## CONFLICT OF INTEREST

None declared.

## Supporting information

Supplementary Material

## Data Availability

The data that support the findings of this study are available from the corresponding author upon request.
