# Supplementary material for: Temporal changes in gene expression and genotype frequency of the ornithine decarboxylase gene in native silverside Basilichthys microlepidotus: Impact of wastewater reduction due to implementation of public policies
Source: Evol Appl. 2020 Jun 22;13(6):1183–94. doi: 10.1111/eva.13000 (PMC7359834; doi:10.1111/eva.13000)
Supplement: Supplementary file 1 — Supplementary Material [file EVA-13-1183-s001.docx]

**SUPPLEMENTARY INFORMATION**

**Temporal changes in gene expression and genotype frequency of the ornithine decarboxylase gene in native silverside *Basilichthys microlepidotus*: impact of**

**wastewater reduction due to implementation of public policies**

**Appendix S1.** Image of the homozygote and heterozygote observed per position in the *odc* gene.

**
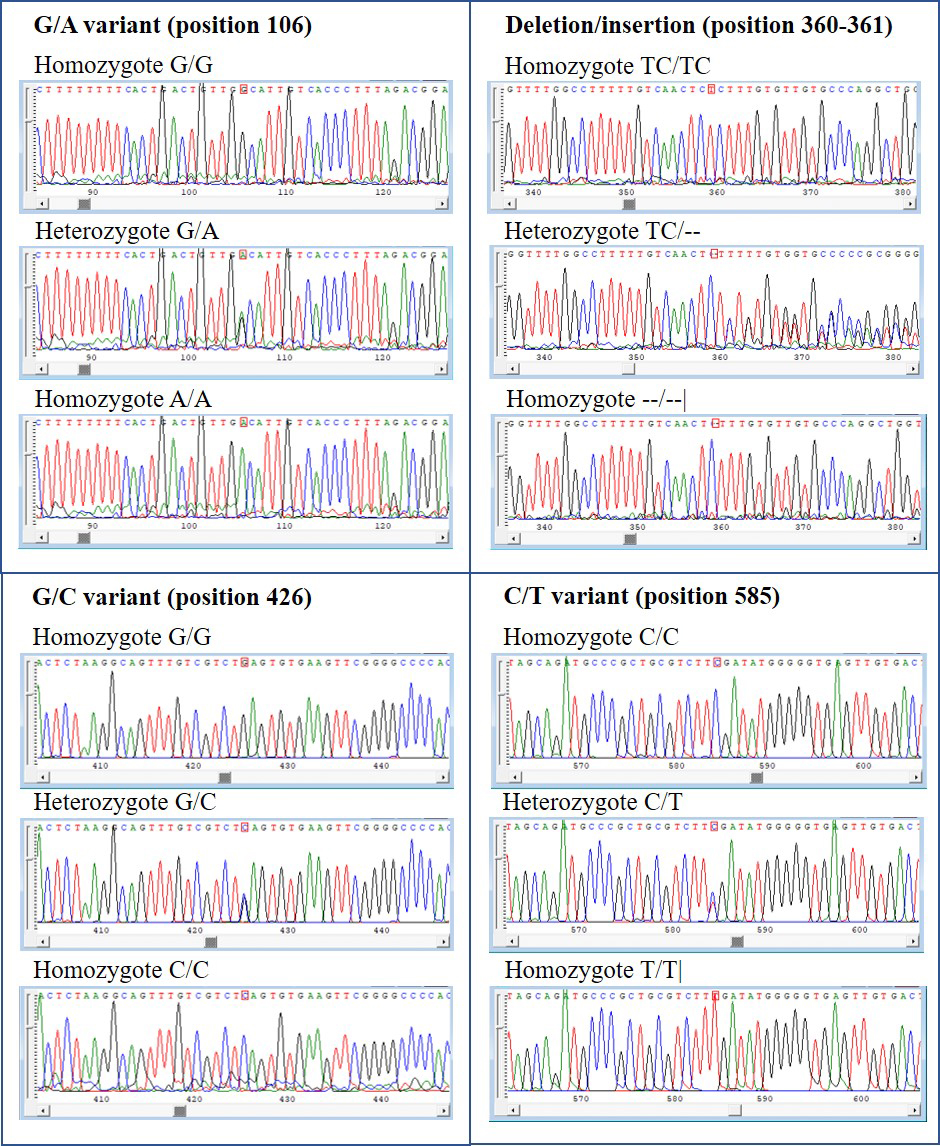
**

**Appendix S2.** Summary of physicochemical data obtained. The average of each physicochemical variable measured in each site and year is showed, standard deviation is showed in parenthesis. MEL: Melipilla; SFM: San Francisco de Mostazal; n: sample size; EC: electrical conductivity; TDS: total dissolved solids; DO: dissolved oxygen; NO_2_: nitrate; NH_4_^+^: ammonium; Na^+^: sodium; K^+^: potassium; Ca^2+^: calcium; Mg^2+^: magnesium.

| Site | Year | n | EC (µS/cm) | pH | TDS (ppm) | DO (µg/L) | NO_2_^-^ (µg/L) | NH_4_^+^ (µg/L) | Na^+^ (mg/L) | K^+^ (mg/L) | Ca^2+^ (mg/L) | Mg^2+^ (mg/L) |
| --- | --- | --- | --- | --- | --- | --- | --- | --- | --- | --- | --- | --- |
| MEL | 2007 | 1 | 1521 | 7.9 | 814 | 5.06 | 0.25 | 40.43 | 117.04 | 1.85 | 141.33 | 12.64 |
| MEL | 2011 | 6 | 1262.5 (176.94) | 7.22 (0.15) | 635 (84.47) | 6.6 (0.85) | 0.1 (0.11) | 33.13 (30.67) | 94.57 (11.56) | 5.46 (0.21) | 159.35 (18.17) | 26.96 (5.3) |
| MEL | 2016 | 3 | 1086.67 (109.7) | 8.18 (0.38) | 1043.67 (105.76) | 11.17 (0.52) | 0.31 (0.19) | 14.67 (6.81) | 181.22 (44.80) | 3.97 (0.68) | 92.67 (7.51) | 9.87 (0.18) |
| MEL | 2017 | 3 | 1260.33 (14.22) | 7.5 (0.08) | 630 (7) | 10.01 (1.38) | 0.0749 (0) | 14.13 (3.03) | 29.06 (1.16) | 4.62 (0.38) | 58.9 (4.22) | 18.68 (0.24) |
| SFM | 2007 | 1 | 392 | 8.33 | 266 | 8.51 | 0 | 39 | 60.12 | 3.3 | 103.6 | 17.42 |
| SFM | 2011 | 5 | 418.6 (20.08) | 6.89 (0.28) | 208.4 (10.69) | 10.2 (1.11) | 0.45 (0.34) | 12.47 (17.08) | 15.8 (3.33) | 1.37 (0.66) | 45.2 (11.41) | 12.36 (0.77) |
| SFM | 2016 | 3 | 396.67 (162.58) | 7.8 (0.46) | 422.33 (110.57) | 5.9 (1.78) | 0.04 (0.01) | 0.09 (0.003) | 99.12 (18.54) | 0.94 (0.05) | 36.89 (7.55) | 10.58 (1.17) |
| SFM | 2017 | 3 | 444.33 (6.03) | 8.58 (0.09) | 223 (2) | 17.7 (0.33) | 0.225 (0.013) | 18.47 (3.8) | 14.65 (1.32) | 1.81 (0.08) | 12.23 (1.17) | 11.1 (0.46) |
|  |  |  |  |  |  |  |  |  |  |  |  |  |

**Appendix S3**. Summary of the microsatellite loci for the different sampling years in the affected (MEL) and unaffected (SFM) sites for *Basilichthys microlepidotus*. Sample size (N). number of alleles (Na). expected heterozygosity (HE). observed heterozygosity (HO). F_IS_ according to Weir and Cockerham (1984). Values with * indicate significant departures to HWE (p < 0.01).

|  |  | MEL | | | |  | SFM | |  |
| --- | --- | --- | --- | --- | --- | --- | --- | --- | --- |
| Locus |  | 2007 | 2011 | 2018 | 2019 | 2007 | 2011 | 2018 | 2019 |
|  |  |  |  |  |  |  |  |  |  |
| Obo01TUF | N | 21 | 19 | 23 | 23 | 23 | 24 | 24 | 23 |
|  | Na | 3 | 4 | 3 | 3 | 3 | 3 | 3 | 3 |
|  | H_E_ | 0.5159 | 0.5693 | 0.5302 | 0.5302 | 0.5189 | 0.4418 | 0.4939 | 0.5397 |
|  | H_O_ | 0.4286 | 0.5263 | 0.5217 | 0.5217 | 0.6087 | 0.3750 | 0.5833 | 0.5217 |
|  | F_IS_ | 0.192 | 0.102 | 0.038 | 0.038 | -0.151 | 0.172 | -0.160 | 0.055 |
|  |  |  |  |  |  |  |  |  |  |
| Obo19TUF | N | 21 | 21 | 24 | 24 | 24 | 24 | 24 | 23 |
|  | Na | 3 | 4 | 6 | 6 | 5 | 7 | 7 | 6 |
|  | H_E_ | 0.1769 | 0.1780 | 0.4757 | 0.4227 | 0.3307 | 0.2995 | 0.4210 | 0.4650 |
|  | H_O_ | 0.1905 | 0.1905 | 0.5000 | 0.4583 | 0.3333 | 0.3333 | 0.4583 | 0.4783 |
|  | F_IS_ | -0.053 | -0.046 | -0.029 | -0.063 | 0.013 | -0.092 | -0.068 | -0.006 |
|  |  |  |  |  |  |  |  |  |  |
|  |  |  |  |  |  |  |  |  |  |
| Obo71TUF | N | 21 | 20 | 24 | 24 | 24 | 22 | 24 | 23 |
|  | Na | 2 | 2 | 2 | 2 | 2 | 3 | 2 | 2 |
|  | H_E_ | 0.4444 | 0.4387 | 0.4132 | 0.3533 | 0.3750 | 0.5217 | 0.4575 | 0.4764 |
|  | H_O_ | 0.2857 | 0.4500 | 0.4167 | 0.3750 | 0.3333 | 0.4091 | 0.5417 | 0.5217 |
|  | F_IS_ |  |  |  |  |  |  |  |  |
|  |  |  |  |  |  |  |  |  |  |
| Odon02 | N | 21 | 22 | 24 | 24 | 24 | 24 | 24 | 23 |
|  | Na | 3 | 1 | 4 | 4 | 4 | 2 | 3 | 3 |
|  | H_E_ | 0.01769 | 0 | 0.2266 | 0.2283 | 0.2292 | 0.0799 | 0.0807 | 0.2316 |
|  | H_O_ | 0.1905 | 0 | 0.250 | 0.1667 | 0.25 | 0.0833 | 0.0833 | 0.2609 |
|  | F_IS_ | -0.053 |  | -0.082 | 0.289 | -0.070 | -0.022 | -0.101 | -0.053 |
|  |  |  |  |  |  |  |  |  |  |
| Odon07 | N | 21 | 21 | 24 | 24 | 24 | 16 | 24 | 23 |
|  | Na | 3 | 3 | 3 | 3 | 3 | 4 | 3 | 3 |
|  | H_E_ | 0.5771 | 0.5748 | 0.5391 | 0.5026 | 0.6120 | 0.6621 | 0.6033 | 0.5822 |
|  | H_O_ | 0.7619 | 0.6667 | 0.5417 | 0.6667 | 0.5417 | 0.5625 | 0.6667 | 0.6087 |
|  | F_IS_ | -0.298 | -0.135 | 0.016 | -0.307 | 0.135 | 0.181 | -0.084 | -0.023 |
|  |  |  |  |  |  |  |  |  |  |
| Odon09 | N | 21 | 22 | 24 | 24 | 24 | 20 | 24 | 23 |
|  | Na | 7 | 7 | 5 | 9 | 7 | 7 | 8 | 6 |
|  | H_E_ | 0.7902 | 0.6870 | 0.6797 | 0.7066 | 0.7031 | 0.7850 | 0.7483 | 0.7164 |
|  | H_O_ | 0.9048 | 0.7273 | 0.7500 | 0.7083 | 0.7917 | 0.7000 | 0.7500 | 0.6957 |
|  | F_IS_ | -0.121 | -0.035 | -0.082 | 0.019 | -0.105 | 0.134 | 0.019 | 0.051 |
|  |  |  |  |  |  |  |  |  |  |
| FIS Total |  | -0.006 | -0.021 | -0.019 | -0.034 | -0.011 | 0.142 | -0.078 | -0.0029 |
|  |  |  |  |  |  |  |  |  |  |

**Appendix S4.** Summary of the *odc* loci for the different sampling years in the affected (MEL) and unaffected (SFM) sites for *Basilichthys microlepidotus*. Sample size (N). number of alleles (Na). expected heterozygosity (HE). observed heterozygosity (HO). F_IS_ according to Weir and Cockerham (1984). Values with * indicate significant departures to HWE (p < 0.01).

|  |  | MEL | | | |  | SFM | |  |
| --- | --- | --- | --- | --- | --- | --- | --- | --- | --- |
| Locus |  | 2007 | 2011 | 2018 | 2019 | 2007 | 2011 | 2018 | 2019 |
|  |  |  |  |  |  |  |  |  |  |
| odc | N | 16 | 17 | 20 | 20 | 19 | 19 | 21 | 19 |
|  | Na | 3 | 4 | 4 | 6 | 6 | 5 | 6 | 6 |
|  | H_E_ | 0.406 | 0.464 | 0.661 | 0.790 | 0.686 | 0.657 | 0.712 | 0.593 |
|  | H_O_ | 0.313 | 0.235 | 0.700 | 0.750 | 0.684 | 0.684 | 0.571 | 0.526 |
|  | F_IS_ | 0.261 | 0.515* | -0.033 | 0.076 | 0.030 | -0.015 | 0.221 | 0.129 |
|  |  |  |  |  |  |  |  |  |  |
